# Supplementary figures and images for: Transcriptomics Profiling of Acer pseudosieboldianum Molecular Mechanism against Freezing Stress
Source: Int J Mol Sci. 2022 Nov 24;23(23):14676. doi: 10.3390/ijms232314676 (PMC9737005; doi:10.3390/ijms232314676)

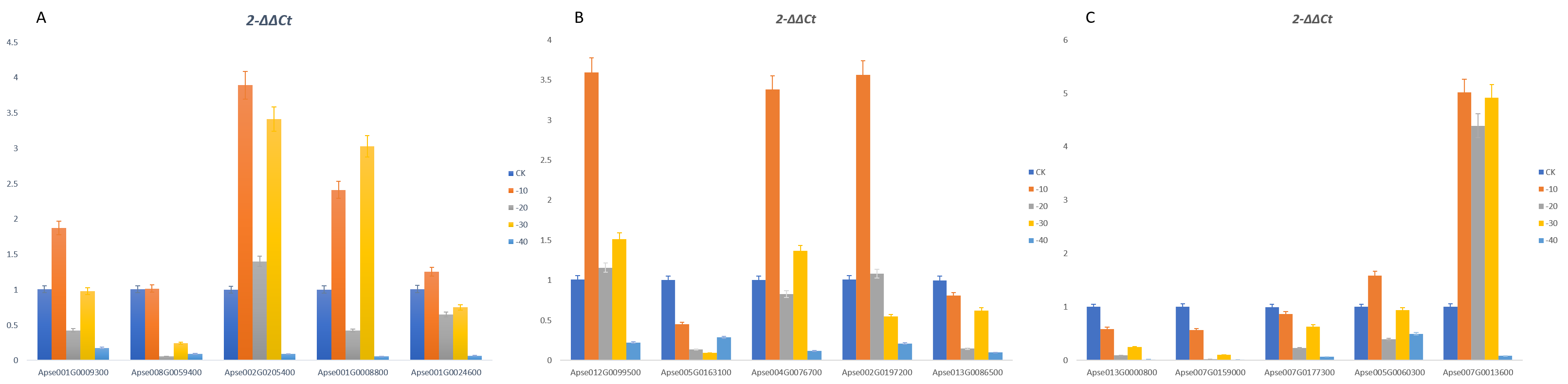

Supplement: Supplementary file 1 [file ijms-23-14676-s001.zip › ijms-1987053-supplementary/Supplementary Figure/FigueS1 Specific primers used for qRT-PCR analysis.png]
